# Curcumin analog A13 alleviates oxidative stress by activating Nrf2/ARE pathway and ameliorates fibrosis in the myocardium of high-fat-diet and streptozotocin-induced diabetic rats

**DOI:** 10.1186/s13098-019-0485-z

**Published:** 2020-01-07

**Authors:** Lanting Xiang, Qiongying Zhang, Chen Chi, Gu Wu, Zhongmin Lin, Jianmin Li, Qianru Gu, Guorong Chen

**Affiliations:** 1 Department of Pathology, The First Affiliated Hospital of Wenzhou Medical University, Wenzhou, Zhejiang People’s Republic of China; 2 Department of Pathology, Sir Run Run Shaw Hospital affiliated To Zhejiang University School of Medicine, Hangzhou, Zhejiang People’s Republic of China

**Keywords:** Diabetes mellitus, Myocardium, Oxidative stress, Curcumin analog

## Abstract

**Background:**

Diabetes mellitus is an important risk factor for cardiomyopathy. Increasing oxidative stress may be one of the main factors of diabetic cardiomyopathy. A13, a newly synthesized curcumin analog, was proved to be superior to curcumin in biological activity. However, little know about how A13 performed in diabetic models. In this study, we evaluated the ability of curcumin analog A13 to alleviate oxidative stress and ameliorate fibrosis in the myocardium, and explore the underlying mechanisms.

**Methods:**

Intraperitoneal injection of streptozotocin (30 mg/kg in 0.1 M sodium citrate buffer, pH 4.5) induced diabetes in high-fat fed rats. The rats were respectively treated with a daily dose of curcumin or A13 via intragastric intubation for 8 weeks. Myocardial tissue sections were stained with hematoxylin–eosin; oxidative stress was detected by biochemical assays; activation of the Nrf2/ARE pathway was detected by Western blot, immunohistochemical staining and RT-qPCR; myocardial fibrosis was identified by Western blot and Masson trichrome staining.

**Results:**

Treatment with curcumin analog A13 reduced the histological lesions of the myocardium in diabetic rats. Curcumin and A13 treatment decreased the malondialdehyde level and increased the activity of superoxide dismutase in the myocardium of diabetic rats. Molecular analysis and immunohistochemical staining demonstrated that dose of 20 mg/kg of A13 could activate the Nrf2/ARE pathway. Molecular analysis and Masson staining showed that curcumin analog A13 treatment significantly ameliorated fibrosis in myocardium of these diabetic rats.

**Conclusion:**

Treatment with curcumin analog A13 protects the morphology of myocardium, restores the MDA levels and SOD activity, activates the Nrf2/ARE pathway and ameliorates myocardial fibrosis in diabetic rats. It may be a useful therapeutic agent for some aspects of diabetic cardiomyopathy.

## Background

Diabetes mellitus (DM), a chronic endocrine metabolic disorder, remains a serious threat to human health. Changing lifestyles and the increasing incidence of non-frail longevity ensure that the incidence of DM continues to rise. Diabetic cardiomyopathy (DCM) is a specific myocardial disease, first described in 1972 [1] in a patient with a history of DM and a wide range of cardiac structural abnormalities. The pathogenesis of DCM is complex, and increasing oxidative stress caused by disorder of glucose metabolism may be one of the key links of DCM [2].

The Nrf2/ARE pathway expresses in various tissue and cells, and acts as the central hub against oxidative stress. Under physiological conditions, NF-E2-related factor 2 (NRF2) binds to the Kelch-like ECH-related protein 1 (KEAP1) in the cytoplasm as a heterodimer. When the body is damaged by oxidative stress or some other agents, the heterodimer dissociates, and the free NRF2 translocates into the nucleus and binds to the antioxidant reaction element (ARE), which increases the expression of the downstream effector proteins [3], like catalase (CAT) and NAD(P)H quinone dehydrogenase 1 (NQO1), to alleviate the oxidative damage and maintain the redox homeostasis.

**Fig. 1:**
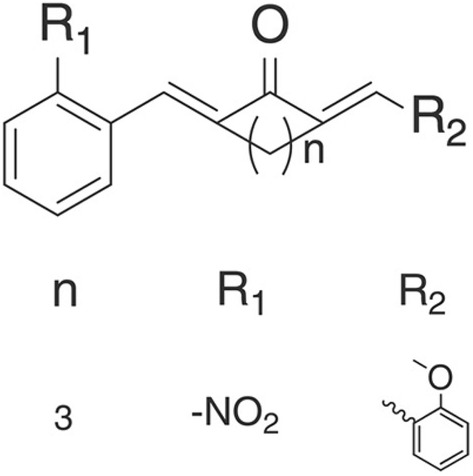
Molecular structure of curcumin analog A13

Curcumin, a yellow-colored pigment extract from the dry roots of turmeric with a formula of C_21_H_20_O_6_, has important economic value and extensive pharmacological effects [4, 5], including anti-oxidative stress, anti-inflammatory, anti-tumor effects. However, curcumin is highly unstable. Increasing numbers of curcumin analogs have been synthesized by removing unstable molecular groups and retaining active ones to overcome its high instability under physiological conditions. As one of the curcumin analogs having the general characteristics of curcumin, A13 (Fig. 1) is superior to curcumin in terms of metabolism and bioavailability [6].

The aim of our study was to examine the microstructural damage caused by diabetes mellitus on the myocardium of rats and the ability of A13 to alleviate oxidative stress by activating the Nrf2/ARE pathway and ameliorate fibrosis in the myocardium of diabetic rats.

## Materials and methods

### Chemicals

Curcumin analog A13 and curcumin was the gifts from professor Guang Liang in Wenzhou Medical University (Wenzhou, China).

### Experimental animals

**Fig. 2:**
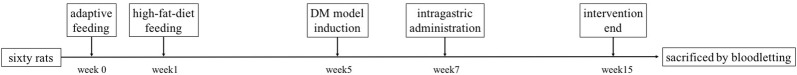
Process of animal experiment

Sixty SPF-rated, male, Sprague–Dawley rats were kept in an experimental animal environment with controlled temperature (23 °C ± 2 °C) and humidity (55% ± 5%). After adaptive feeding for 1 week, ten rats were randomly assigned to a non-diabetic control group (NC), and the remainder formed the experimental group. The experimental group was fed with high-fat food [7] for 4 weeks while the NC group received a standard diet. After 4 weeks, the experimental group was injected intraperitoneally with streptozotocin (STZ) (30 mg/kg in 0.1 M sodium citrate buffer, pH4.5) to induce diabetes whereas the NC group was injected with the carrier vehicle alone. Rats (n = 42/50) with a blood glucose level of ≥ 16.7 mmol/L were considered diabetic. These diabetic rats were further randomly divided into diabetes mellitus group (DM, n = 12), curcumin treatment group (CUR, n = 10), high-dose A13 treatment group (H-A13, n = 10) and low-dose A13 treatment group (L-A13, n = 10). The CUR group and H-A13 group were respectively treated with a daily dose [6] of curcumin or A13 (20 mg/kg body weight) for 8 weeks via intragastric administration; the L-A13 group was treated with A13 (10 mg/kg body weight). At the same time, the other groups were treated with vehicle in the same dose. At the end of the experiment all anesthetized rats were sacrificed by bloodletting, and relevant specimens were taken for further study. The process of animal experiment is shown in Fig. 2.

### Histopathologic evaluation

The myocardial specimens were fixed in 10% buffered formalin, embedded in paraffin, and sliced into 3.5 μm sections. Hematoxylin–eosin (HE) staining and Masson staining were performed to evaluate histological changes, including the status of myocardial cells and fibrosis. Image J 1.50i (National Institute for Health, Maryland, USA) for semi-quantitative analysis of myocardial collagen volume (CVF).

### Biochemical assays

The protein content of the supernatant in myocardial homogenate was determined by BCA method [8]. Level of malondialdehyde (MDA) and activity of superoxide dismutase (SOD) of myocardium were measured with the thiobarbituric acid method and hydroxylamine methods, respectively.

### Immunohistochemical (IHC) staining

NRF2 and CAT immunostaining were performed using a standard protocol [9] with polyclonal anti-NRF2 antibody (product code ab31163, 1:250 dilution; Abcam, plc, Cambridge, UK) and monoclonal anti-CAT antibody (product code ab76110, 1:250 dilution; Abcam, plc, Cambridge, UK) respectively. Appropriate negative and positive controls were included in each batch of immunohistochemical staining.

### Western blot analysis

Nucleoprotein extraction was conducted as described in the nuclear and cytoplasmic protein extraction kit (product code: P0028; Beyotime Biotechnology, Inc, Shanghai, China) and total protein extraction was conducted using a standard protocol [10]. For Western blot analysis, an equal amount of protein (20 μg) from each myocardial sample was separated on a 12% sodium dodecyl sulfate–polyacrylamide gel and transferred to a polyvinylidene difluoride membrane. The membranes were blocked in 5% milk to prevent nonspecific binding at room temperature for 1.5 h and then incubated at 4 °C overnight with primary antibodies. The primary antibodies included anti-COL1A2 antibody (product code BS1530, 1:500 dilution; Bioworld Technology, Inc, St. Louis Park, MN, USA), anti-TGF-β1 antibody (product code ab25121, 1:2000 dilution; Abcam, plc, Cambridge, UK), anti-NRF2 antibody (product code ab31163, 1:1000 dilution; Abcam, plc, Cambridge, UK), anti-CAT antibody (product code ab76110, 1:1000 dilution; Abcam, plc, Cambridge, UK) and anti-NQO1 antibody (product code ab80588, 1:10,000 dilution; Abcam plc, Cambridge, UK). The membranes were then incubated with appropriate, horseradish peroxidase (HRP)-conjugated secondary antibody (1:1000 dilution) for 1 h, and visualized the results using enhanced chemiluminescence reagents through ChemiDoc™ XRS + system (Bio-Rad Laboratories, Inc, CA, USA). Glyceraldehyde 3-phosphate dehydrogenase (GAPDH) was used as an internal control for total protein loading to normalize each sample, while LAMIN B1 was used as the internal control for nucleoprotein loading.

### RNA isolation and analysis by RT-qPCR

Myocardial specimens were homogenized, and total RNA isolated using TRIzol reagent (Thermo Fisher Scientific, Walther, MA, USA). The concentration and purity of RNA was determined by multifunctional enzyme assay. Reverse transcription (RT) to cDNA was performed with 500 ng total RNA and 5 × PrimeScript RT Master Mix (product code: RR036A, Takara Bio, Inc, Japan). The RT mixture was sequentially incubated at 37 °C for 15 min and 85 °C for 5 s before quick chilling at -20 °C. The qPCR reactions were performed in duplicate in a fluorescence quantitative PCR detection system (product code: CFX96, Bio-Rad Laboratories, Inc, CA, USA) using 2 × ChamQ SYBR qPCR Master Mix (product code: Q311-02, Vazyme Biotech Co., Ltd, Nanjing, China), 10 μM of forward and reverse primer and template cDNA. The qPCR mixture was firstly incubated at 95 °C for 30 s as the predenaturation, and then sequentially incubated at 95 °C for 10 s, 60 °C for 30 s, 40 cycles in total. 2^−△△Ct^ was calculated to analyze the relative mRNA expression of target genes. Primers used for the amplification of the products were listed in Table 1.

**Table 1:** Primers for myocardium genes

Gene	Forward and reverse primers
*Gapdh*	F: 5′-CCTTCCGTGTTCCTACCC-3′
	R: 5′-AAGTCGCAGGAGACAACC-3′
*Nrf2*	F: 5′-ACTGTCCCCAGCCCAGAGGC-3′
	R: 5′-CCAGGCGGTGGGTCTCCGTA-3′
*Cat*	F: 5′-ACTGAAGATGGTAACTGGGA-3′
	R: 5′-ATGGATAAAGGATGGAAACA-3′
*Nqo1*	F: 5′-ACTACGATCCGCCCCCAACTTCTG-3′
	R: 5′-CTTCGGCTCCCCTGTGATGT-CGT-3′

### Statistical analysis

Data were presented as the mean mean ± SD and analysed using SPSS 22.0 (IBM, New York, USA) software. Image Lab 5.2.1 (Bio-Rad, California, USA) was used to analyze the Western blots. Graph Pad Prism 6.0 (Graphpad Software, San Diego, USA) was used to draw bar chart. Comparisons were performed by one-way analysis of variance (ANOVA) and Bonferroni-corrected *t* test for the different groups. Differences were considered significant at p < 0.05.

## Results

### Role of curcumin analog A13 on myocardium morphology of diabetic rats

**Fig. 3:**
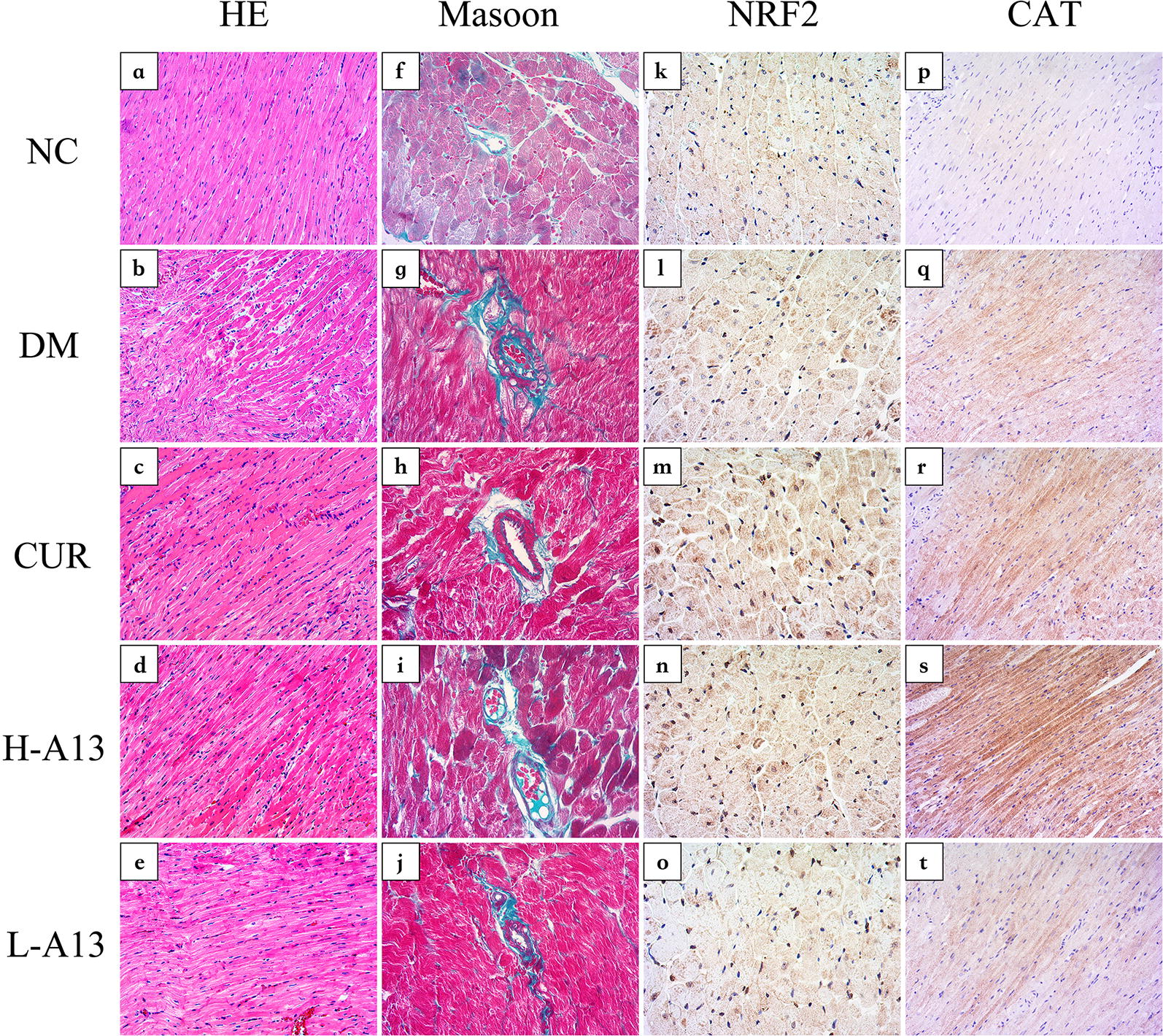
Representative microscopical images of myocardium tissue sections from each groups. Tissue sections were staind with HE staining (**a**–**e**, ×200 magnification), masson staining (**f**–**j**, ×400 magnification), NRF2 IHC staining (**k**–**o**, Nrf2: ×400 magnification) and CAT IHC staining (**p**–**t**, ×200 magnification)

Upon HE staining and under a microscope, the myocardial cells from the rats of NC group were slender and orderly, and the cytoplasm was dyed red and evenly colored (Fig. 3a); the myocardium from DM group, however, demonstrated hypertrophied, myocardial cells, some of which were contracted, wavy and even ruptured (Fig. 3b). Notably, treatment with curcumin analog A13 restored the morphology of the myocardial cells (Fig. 3c–e) to an extent comparable to that of control group and the recovery in the H-A13 group was more significant, suggesting that curcumin analog A13 may protect the myocardium of rats against structural damage caused by hyperglycemia.

### Effect of curcumin analog A13 on anti-oxidative stress

**Fig. 4:**
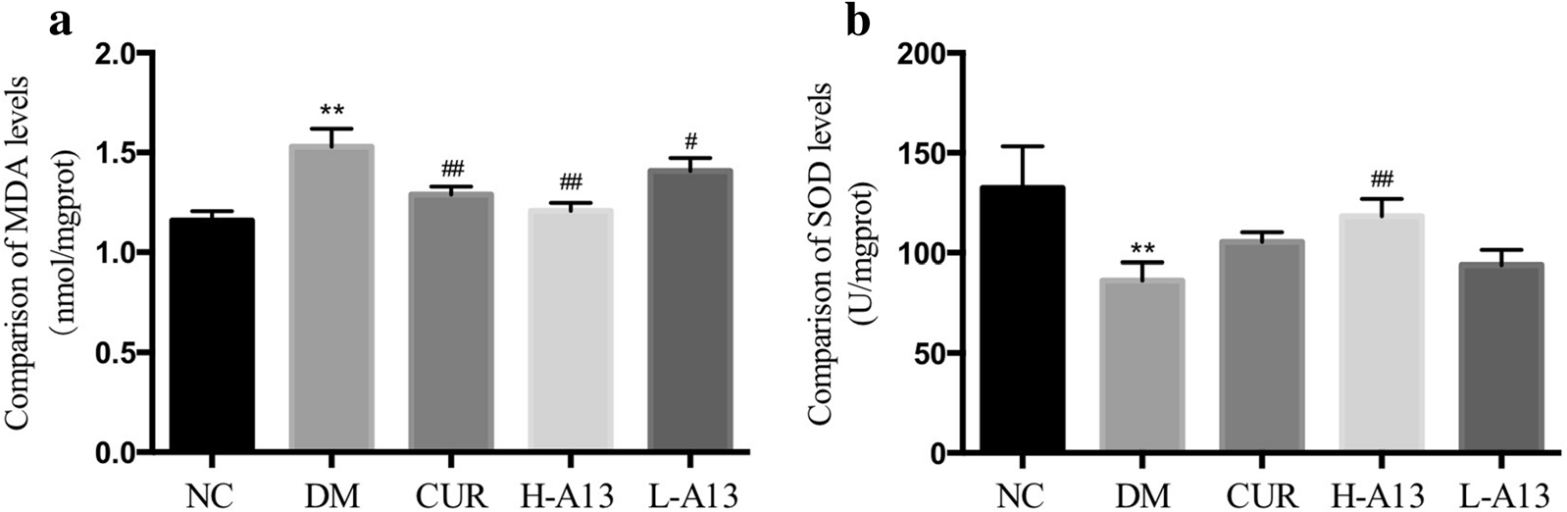
Effect of curcumin analog A13 on oxidative stress-related parameters. MDA level (**a**) and activity of SOD (**b**). All data are presented as mean ± SD. **p < 0.05 versus NC group; ^#^p < 0.05, ^##^p < 0.01 versus DM group

The MDA level was significantly increased and the SOD activity was remarkably reduced in the myocardium of DM group when compared with NC group. By contrast, treatment with curcumin or A13 remarkably reversed the trend by reducing the MDA level and increasing SOD activities in the myocardium of diabetic rats. However, changes of SOD activity in CUR and L-A13 groups were not significant (Fig. 4).

### Ability of curcumin analog A13 to activate the Nrf2/ARE pathway in diabetic rats

**Fig. 5:**
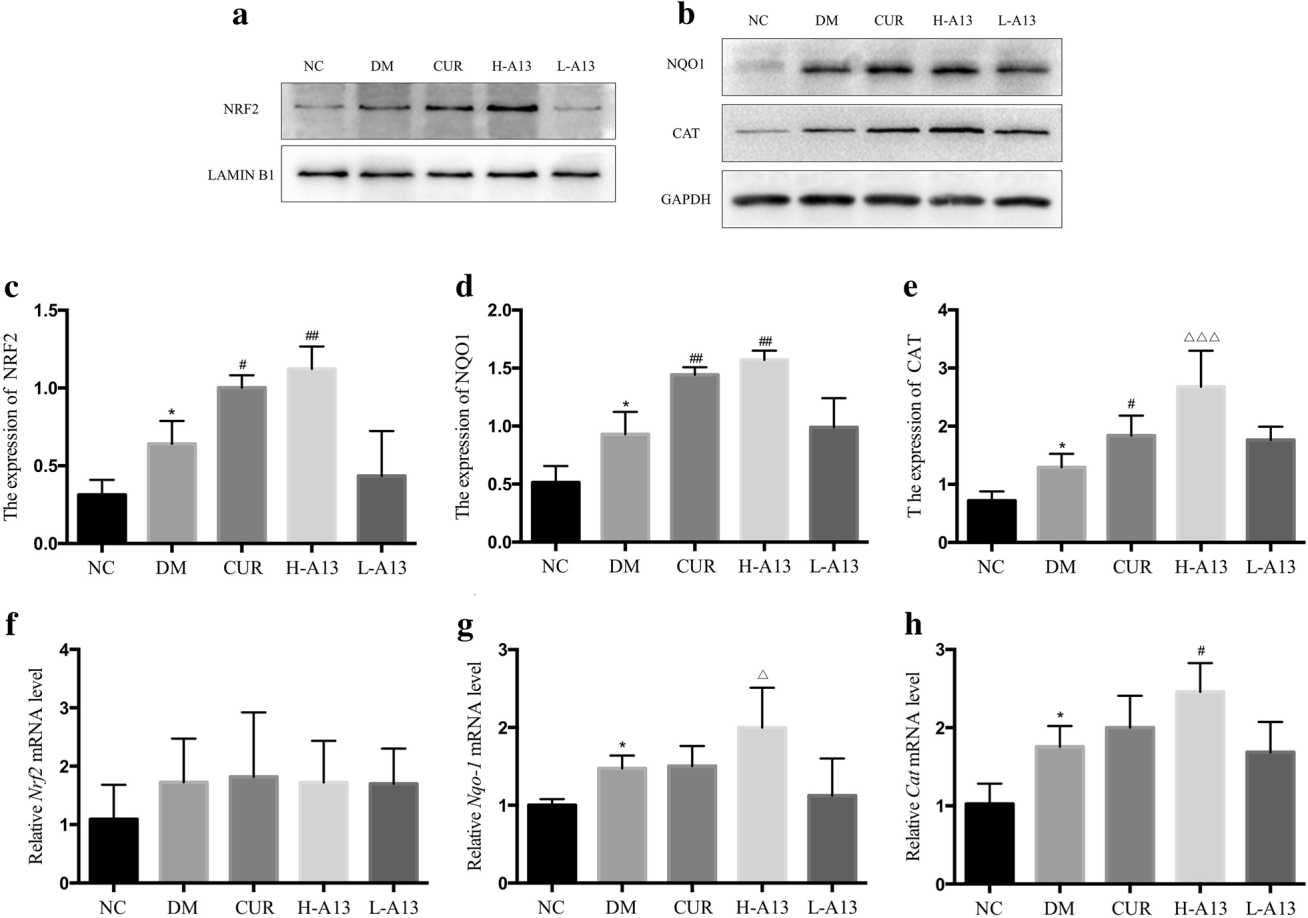
Ability of curcumin analog A13 to activate the Nrf2/ARE pathway as detected by Western blot and RT-qPCR. Level of NRF2 in nucleus (**a**, **c**) and relative mRNA expresion of *Nrf2* (f); Expression of NQO1 and CAT (**b**, **d**, **e**) and relative mRNA expression of *Nqo1* and *Cat* (**g**, **h**). All data are presented as mean ± SD. *p < 0.05 versus NC group; ^#^p < 0.05, ^##^p < 0.01 versus DM group; ^△^p < 0.05, ^△△^p < 0.01 versus DM and CUR groups

In the DM group, the protein level of NRF2 by Western blot was significantly increased in myocardial nuclei in contrast with NC group. After treatment with curcumin or high-dose of A13, the levels of NRF2 significantly increased but there was no significant change in the L-A13 group (Fig. 5a, c). Expression levels of CAT and NQO1 in cytoplasm showed the same trend as the NRF2 level (Fig. 5b, d, e). Besides the Western blot, the RNA expression of *Nrf2*, *Nqo1* and *Cat* in myocardium was also examined by RT-qPCR, and there was no significant difference between the five groups, while the expression trend of *Nqo1* and *Cat* was similar to that of the protein level, but not exactly the same (Fig. 5f–h).

We also used IHC staining to examine their in situ distribution and expression. NRF2 positive staining was localized in nucleus and cytoplasm. NRF2 staining was weakly positive and uniformly distributed throughout the cytoplasm for the control group (Fig. 3k) compared to the clustered staining in the cytoplasm and sometimes scattered in the nucleus for the DM group (Fig. 3l). NRF2 staining in the cytoplasm enhanced, forming a ring around the nucleus with some positive staining in the nucleus in samples of rats treated with curcumin or high-dose of A13 (Fig. 3m, n). The change was not significant in the low-dose A13 group (Fig. 3o). CAT staining was localized in the cytoplasm, a pattern consistent with changes in the Western blot (Fig. 3p–t).

### Curcumin analog A13 to ameliorate fibrosis in diabetic rats

**Fig. 6:**
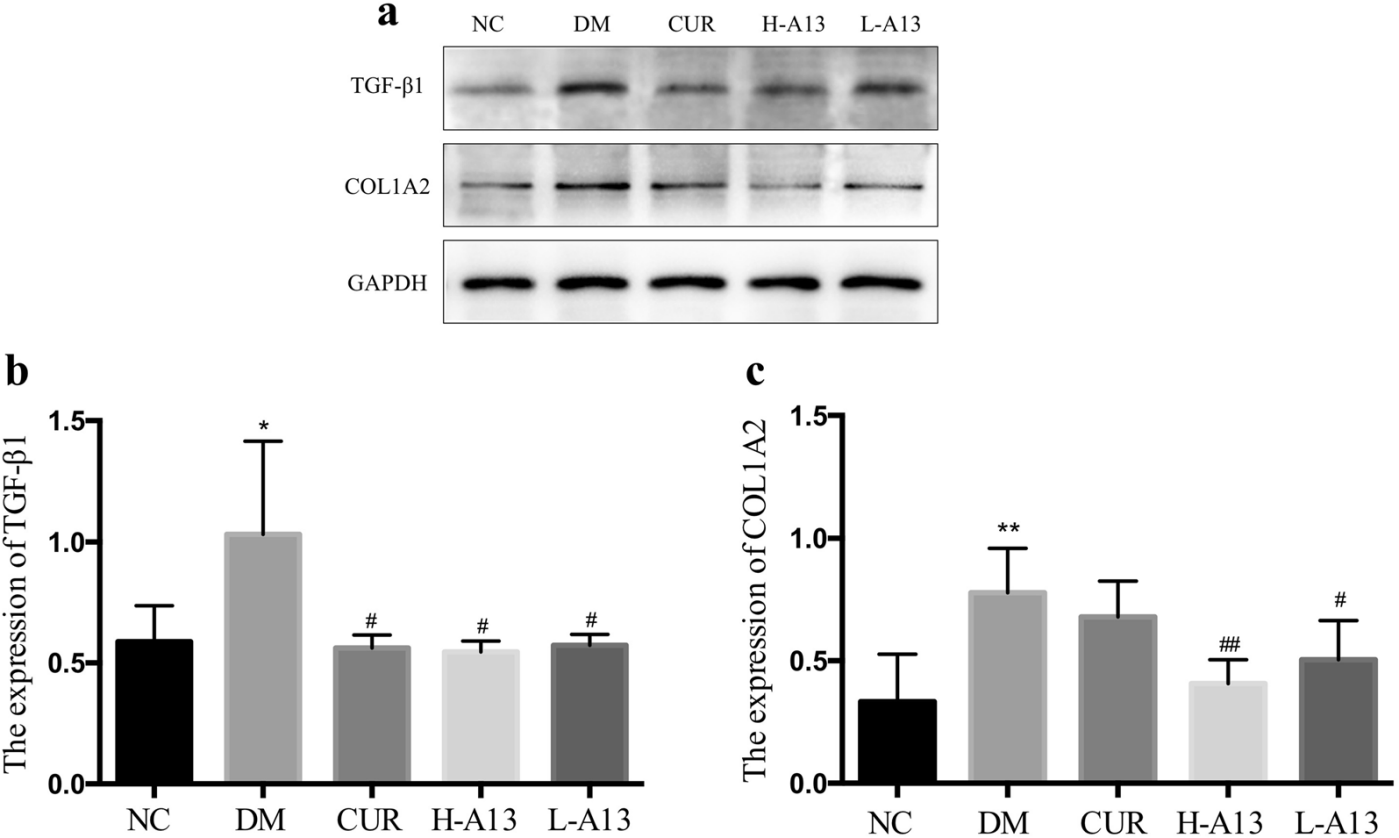
Expression of TGF-β1 and COL1A2 were detected by Western blot. Expression of TGF-β1 and COL1A2 (**a**–**c**). All data are presented as mean ± SD. *p < 0.05, **p < 0.01 versus the NC group; ^#^p < 0.05, ^##^p < 0.01 versus the DM group

By Western blot, the expression of Transforming growth factor-β1 (TGF-β1) and Collagen-type I-alpha 2 (COL1A2) in the myocardium were significantly increased for the DM group (Fig. 6) in compared to controls. At the end of the curcumin or A13 treatment, TGF-β1 expression and COL1A2 expression were reduced to varying extent, while no significant difference was found in the COL1A2 expression for the CUR group (Fig. 6).

**Fig. 7:**
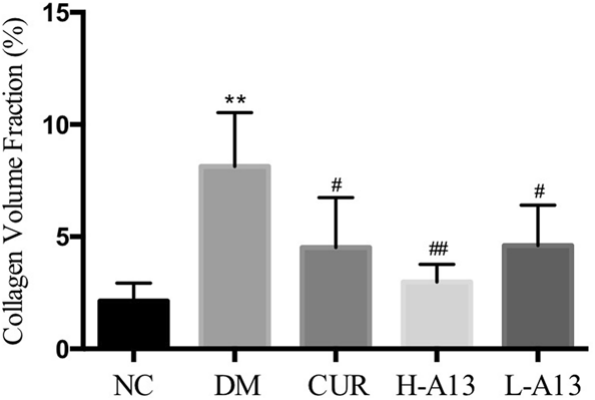
CVF of myocarium from each groups. All data are presented as mean ± SD. **p < 0.01 versus the NC group; ^#^p < 0.05, ^##^p < 0.01 versus the DM group

Of note, the changes were similar by Masson staining of myocardium sections. Hyperplasia and deposition of bright, green-stained collagen was observed in the cardiac interstitial and vascular walls for the DM group, compared to smaller amounts of collagen deposition for the control group. Moreover, curcumin or A13 treatment significantly ameliorated the hyperplasia and deposition of collagen in the myocardium of diabetic rats (Fig. 3f–j). The CVF of DM group was significantly increased when compared to the NC group. In contrast, the CVF remarkably decreased after treating with curcumin or A13, especially in H-A13 group (Fig. 7).

## Discussion

This study shows that treatment with curcumin or its analogs can ameliorate some of cardiac dysfunctions caused by STZ-induced diabetes, such as glucose metabolism, oxidative stress and myocardial fibrosis. The Nrf2/ARE pathway plays an important role in a series of oxidative stress mediated diseases [11]. During oxidative stress [11], NRF2/KEAP1 dimer dissociates; NRF2 enters the nucleus, binds with ARE, and promotes expression of antioxidant protein and phase II detoxification enzyme to maintain the homeostasis of oxidative-reductive microenvironment. Curcumin can relieve oxidative stress through scavenging ROS [12–14], with direct antioxidant capacity. Indirectly, curcumin can covalently modify the cysteine residue of KEAP1 [15], which promotes the dissociation of NRF2/KEAP1 dimer and the nuclear translocation of NRF2.

In our study, NRF2 presented nuclear translocation in the hyperglycemic state, and its nuclear content continued to increase after treatment with curcumin or A13. Downstream expression of protein CAT and NQO1 was consistent with it, while the mRNA expression trend was not completely consistent, which may be related to the spatio-temporal interval from transcription to translation [16]. Combined with the experimental results of our study, A13 may exert antioxidant stress effect by activating the Nrf2/ARE pathway and promoting nuclear translocation of NRF2.

Hyperglycemia may increase the ROS level in myocardial myocytes, and too much ROS will trigger oxidative stress and cause lipid peroxidation, leading to the production of cytotoxic MDA. In contrast to the indirect representation of the degree of oxidative stress damage by MDA, SOD can function to scavenge oxygen radicals, so its activity and content could directly reflect the overall status against oxidative stress. Studies have shown that the oxidative stress level in multiple organs, including the myocardial cells, of the diabetic rats was overwhelming, as indicated by the increasing level of MDA and decreasing activity of SOD [7, 17, 18]. Consistent with previous findings, treatment of curcumin or A13 in our study decreased the oxidative stress levels, which may relate to its activation of the Nrf2/ARE pathway.

Studies have shown that TGF-β1 promotes mRNA expression of type I collagen in fibroblasts [19, 20], stimulates the proliferation of cardiac fibroblasts and collagen synthesis [21]. Myocardial fibrosis is characterized by myocardial stromal remodeling; hyperglycemia, oxidative stress and excessive accumulation of ROS may be important factors in the formation and development of myocardial fibrosis [22, 23]. Researchers have shown that excessive ROS induced by oxidative stress can up-regulate the activity of MMP family proteins such as MMP-2, activate mitogen-activated protein kinase signaling pathway [24] and inactivate NO secreted by vascular endothelial cells [25], thus causing metabolic disorders in myocardial cells and promoting synthesis and secretion of collagen. In our study, expression of TGF-β1 and COL1A2 was higher in diabetic rats than normal rats and decreased after curcumin or A13 treatment, which was supported by results of Western blot, qPCR and Masson staining. Further study is warranted to determine the mechanisms how oxidative stress causes fibrosis.

## Conclusion

This study is first to compare curcumin analog A13 with curcumin, we found that curcumin analog A13 has better performance than curcumin, which can protect the myocardium in diabetic rats by activating Nrf2/ARE pathway and ameliorates fibrosis. Our study may provide a new potential therapeutic idea and target for DCM.

## Data Availability

The datasets used and analysed during the current study are included in this published article.

## Abbreviations

DM: diabetes mellitues
DCM: diabetic cardiomyopathy
NRF2: NF-E2-related factor 2
KEAP1: Kelch-like ECH-related protein 1
ARE: antioxidant reaction element
CAT: catalase
NQO1: NAD(P)H quinone dehydrogenase 1
NC: non-diabetic control
CUR: curcumin
H-A13: high-dose A13 treatment
L-A13: low-dose A13 treatment
CVF: myocardial collagen volume
STZ: streptozotocin
HE: hematoxylin–eosin
MDA: malondialdehyde
SOD: superoxide dismutase
IHC: immunohistochemistry
TGF-β1: transforming growth factor-β1
COL1A2: collagen-type I-alpha 2
GAPDH: glyceraldehyde 3-phosphate dehydrogenase
RT: reverse transcription
